# Personalized medicine, scientific revolution or self-serving illusion: viewpoint from EORTC-PAMM Group

**DOI:** 10.1016/j.esmoop.2026.108576

**Published:** 2026-09-23

**Authors:** V. Debien, A. Geraud-Cremieux, M. Le Grand, D. Carbone, I. Garajova, C. Pecoraro, E. Pasquier, G.J. Peters, E. Giovannetti, M. Centanni, J. Ciccolini

**Affiliations:** 1 Early Phase Clinical Trials Unit, Institut Bergonié, Bordeaux, France; 2 Medical Oncology Department, Institut Paoli-Calmette, Marseille, France; 3 Centre de Recherche en Cancérologie de Marseille (CRCM), Aix-Marseille Université (U105), Inserm (UMR1068), CNRS (UMR7258), Institut Paoli Calmettes, REMAP4Kids Team, Translate-IT department, SouthROCK Center of Excellence in Pediatric Oncology Research, Marseille, France; 4 Department of Biological, Chemical, and Pharmaceutical Sciences and Technologies (STEBICEF), University of Palermo, Palermo, Italy; 5 University Hospital of Parma, Medical Oncology Unit, University Hospital of Parma, Parma, Italy; 6 Department of Biochemistry, Medical University of Gdansk, Gdansk, Poland; 7 Department of Oncology, Amsterdam UMC, Vrije Universiteit Amsterdam, Amsterdam, The Netherlands; 8 Cancer Biology and Immunology, Cancer Center Amsterdam, Amsterdam, The Netherlands; 9 Cancer Pharmacology Laboratory, Fondazione Pisana per la Scienza, San Giuliano, Pisa, Italy; 10 Department of Pharmacy, Uppsala University, Uppsala, Sweden; 11 COMPO Team, Aix Marseille University, INRIA, CNRS, INSERM, Institut Paoli-Calmettes, CRCM, Marseille, France; 12 Biogenopole, La Timone University Hospital of Marseille, Assistance Publique-Hôpitaux de Marseille (AP-HM), Marseille, France

**Keywords:** pharmacokinetic/pharmacodynamic modeling, precision oncology, real-world evidence, therapeutic drug monitoring, tumor-agnostic therapy

## Abstract

Personalized medicine has redefined oncology by tailoring treatments to molecular and clinical characteristics. However, despite remarkable advances in biomarker discovery and targeted therapy, the integration of pharmacological principles into precision oncology remains limited. The resulting discrepancy between biological innovation and therapeutic optimization contributes to high attrition rates in drug development, inconsistent real-world efficacy, and escalating financial toxicity. The European Organisation for Research and Treatment of Cancer Pharmacology and Molecular Mechanisms Group explored how pharmacology can bridge the gap between molecular precision and clinical reality. Although understanding mechanisms of action is a critical first step, the subsequent translation of this knowledge into individualized dosing and scheduling strategies and rational drug combinations is equally essential. Achieving this requires the use of quantitative tools, such as pharmacokinetic/pharmacodynamic modeling, therapeutic drug monitoring, and real-world evidence (RWE). These patient-centered approaches enable continuous learning from patient data, refine our understanding of exposure–response relationships, and support adaptive treatment strategies across diverse populations. We also discuss the rise of tumor-agnostic approvals, which transcend histological boundaries yet expose disparities in national regulatory and reimbursement frameworks. Ensuring equitable access to mechanism-driven therapies requires harmonized policies integrating pharmacological, clinical, and ethical perspectives. True personalization in oncology requires moving beyond target selection to delivering the right dose to the right patient at the right time. Embedding holistic pharmacology approaches into the design, evaluation, and postmarketing use of anticancer therapies will improve efficacy, minimize toxicity, and promote value-based care. Integrating mechanistic understanding with quantitative pharmacology and RWE represents the next frontier of precision oncology, transforming innovation into sustainable, patient-centered benefit.

## Highlights

• Personalized oncology requires pharmacology-driven dosing beyond molecular target selection alone.
• PK/PD modeling and TDM enable patient-specific dose optimization in cancer care.
• Modern anticancer drugs expose the limits of empirical dosing and one-size-fits-all strategies.
• Integrating pharmacology, biology, and policy is key to equitable and sustainable precision oncology.

## Introduction

Personalized medicine is reshaping oncology by moving away from the outdated ‘one-drug-fits-all’ paradigm toward therapies tailored to the molecular, genetic, and clinical characteristics of each patient. This transformation, rooted in decades of biological discovery and pharmacological innovation, has already revolutionized cancer care. Over the last two decades, the oncology field has witnessed the emergence of oral targeted therapies, chimeric antigen receptor T cells, immune checkpoint inhibitors, mRNA vaccines, antibody–drug conjugates (ADCs), and bispecific antibodies.^1^ These advances have redefined and often improved treatment expectations for many tumor types yet the implementation of precision medicine in routine practice remains incomplete. Indeed, if the one-drug-fits-all paradigm has been replaced by therapies guided by tumor molecular features, further progress is needed to shift from the one-size-fits-all model to the ‘one patient, one dose’ approach.

Despite major breakthroughs, the clinical success rate of oncology drug development remains among the lowest across therapeutic areas, with <5% of agents entering trials ultimately reaching regulatory approval.^2^ The main causes of failure include insufficient efficacy, unanticipated toxicity, and limited translatability of early-phase results to heterogeneous real-world populations.^3^ This is aggravated by the current trend of replacing traditional phase II studies by phase I-II trials where the recommended phase II dose is tested in a limited expansion cohort. Additionally, as many innovative drugs in oncology are now designated ‘breakthrough technology’, they are frequently approved after a fast-track process, skipping comparative phase III trials. Consequently, adverse events or unexpected pharmacodynamic (PD) effects are less likely to be detected during clinical development due to the shortened study duration and reduced patient numbers, thereby lowering the probability of observing rare events in outlier individuals. At the same time, the small proportion of drugs that succeed are often associated with substantial costs, while still only being efficacious in a subset of patients, raising concerns about financial toxicity and equitable access to care.^4^ These realities underscore a fundamental gap between innovation and patient benefit. Some efforts, such as the Project Optimus launched by the Food and Drug Administration (FDA), aim to include more rationale in dose selection, i.e. by running comparative trials between two doses before starting a phase II trial.^5^^,^^6^ However, several challenges remain, such as poor integration of patient-reported outcomes (PROs), which can underestimate safety issues and possibly result in eventual overestimation of the maximum tolerated dose (MTD) and poor tolerance once the drug is approved.

Understanding and applying pharmacological principles is central to closing this gap. Personalized oncology should focus not only on identifying actionable molecular targets but also on how drugs behave across patients, how exposure relates to response and toxicity, and how to achieve optimal dosing across populations. Connecting pharmacology with clinical oncology, from mechanism discovery to dose optimization, will be vital to improve efficacy, safety, and value.

In this article, the European Organisation for Research and Treatment of Cancer Pharmacology and Molecular Mechanisms (EORTC-PAMM) Group summarizes how pharmacological science can enhance the implementation of personalized medicine in oncology. We examine how mechanistic understanding, pharmacokinetic (PK) and PD modeling, and real-world evidence (RWE) can help to design smarter trials, rational dosing, and adaptive treatment strategies. The goal is to demonstrate that true personalization extends beyond tumor biology: it requires integrating biological, pharmacological, and patient-centered perspectives to deliver therapies that are not only innovative but also equitable and sustainable.

## Definition of personalized medicine from a pharmacological point of view

The terms ‘personalized medicine’ and ‘precision medicine’ are often used interchangeably yet they differ subtly in scope and intent. Whereas ‘precision medicine’ emphasizes clustering of patients based on molecular alterations and predictive biomarkers, ‘personalized medicine’ extends further, considering an individual’s pharmacological profile, comorbidities, physiological parameters, and lifestyle factors that influence treatment response (Figure 1).^7^ In oncology, these two dimensions are inseparable: effective personalization requires both biological precision and pharmacological adaptation in a patient-centered global approach.

**Figure 1:**
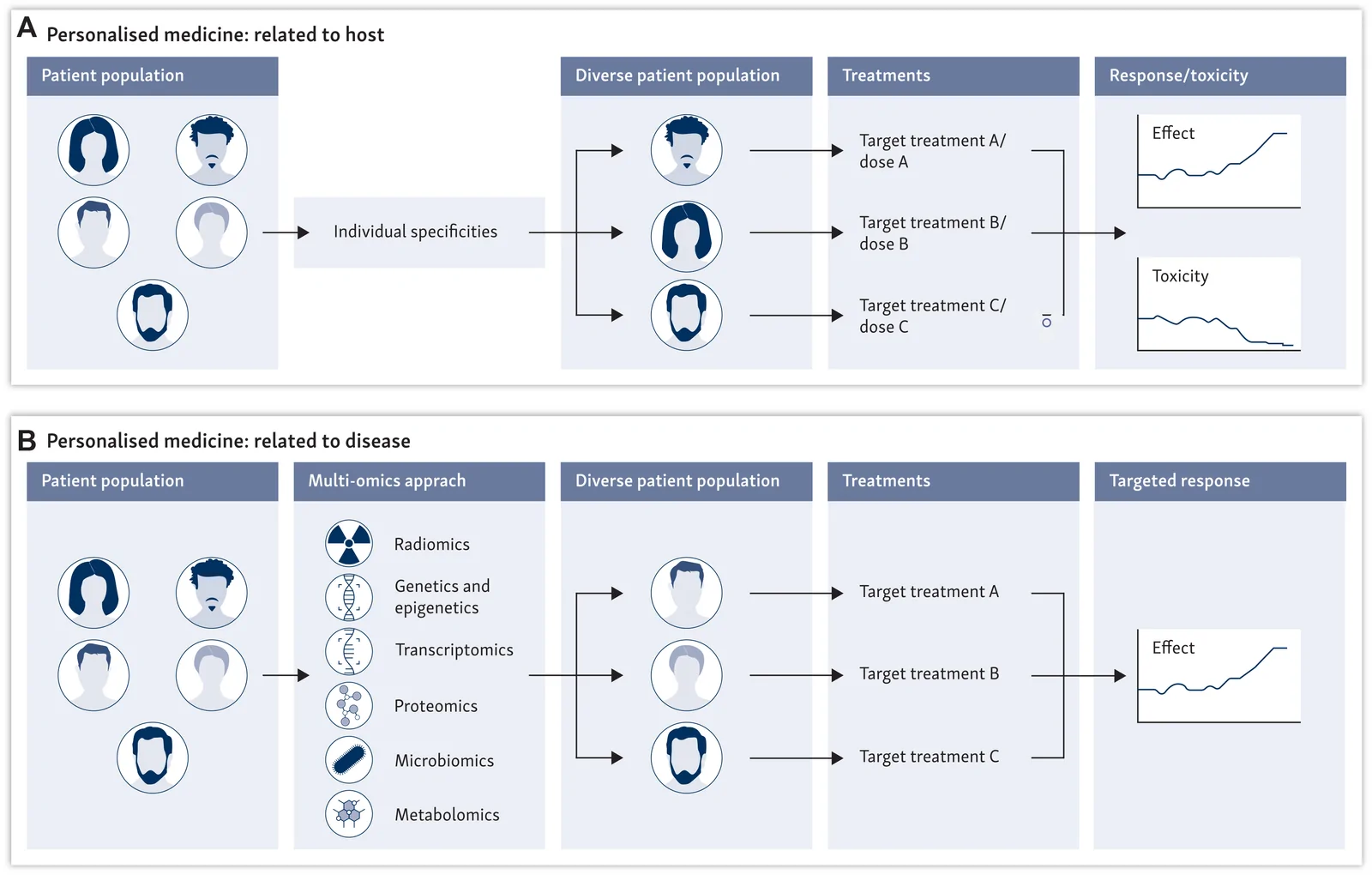
**Approaches to personalizing cancer treatment based on patient and disease characteristics.** Personalized medicine in oncology can be broadly categorized into two complementary strategies: personalization related to the patient background and characteristics and personalization related to specific molecular diseases. (A) Host-related approaches consider individual patient characteristics—such as organ function, comorbidities, pharmacogenetics, and immune status—that influence drug metabolism, distribution, and tolerability. These factors guide selecting and adjusting treatment regimens, including dose modifications, intended to optimize therapeutic efficacy while minimizing toxicity. (B) Disease-related personalization focuses on the molecular and biological features of the tumor. By integrating data from radiomics, genomics, transcriptomics, proteomics, the microbiome, and metabolomics, this approach enables the stratification of patients into subgroups based on tumor-specific vulnerabilities. This facilitates the selection of targeted therapies and supports more precise treatment decisions. Together, these strategies aim to tailor treatment to the patient and their disease, improving outcomes and reducing adverse effects. Figure created with BioRender.

From a pharmacological standpoint, personalized medicine aims to maximize therapeutic benefit while minimizing toxicity by understanding how drugs behave in ‘real’ patients. Drug exposure and response are shaped by multiple host-related determinants: organ function, age, sex, genetic polymorphisms, concomitant medications, and even nutrition or circadian rhythm. Recognizing this variability is essential to ensure that the right dose reaches the right target in the right patient.

Despite major advances in biomarker-driven therapy, implementation in clinical trials often remains confined to highly selected patient populations in specialized centers. Stringent inclusion criteria, focusing on organ function and performance status, reduce interindividual variability but also limit the generalizability of findings. As a result, early-phase studies frequently define a ‘recommended’ dose that may be suboptimal for broader real-world populations, such as elderly individuals, polymedicated patients, or those with comorbidities. Indeed, patients aged >65 years represent up to 60% of patients with cancer, whereas they account for only 22%-32% of clinical trial enrollments.^8^ The underrepresentation of these groups, who constitute the majority of patients in everyday oncology, contributes to unforeseen toxicities and inconsistent efficacy once drugs enter clinical practice.^9^

Integrating pharmacology into precision oncology offers concrete solutions to this translational gap. Therapeutic drug monitoring (TDM), population PKs, and model-informed precision dosing (MIPD) are tools that can individualize therapy by bridging dosing, exposure, efficacy, and toxicity. In routine care, TDM of oral tyrosine kinase inhibitors and other targeted therapies already helps clinicians to identify inadequate exposure or excessive toxicity.^10^^,^^11^ Yet, despite a strong pharmacological rationale, only a few anticancer agents currently have validated exposure–effect relationship (EER) and exposure–safety relationship (ESR) thresholds to guide clinical practice.^12^ For example, immune checkpoint inhibitors, such as the anti-cytotoxic T-lymphocyte-associated protein 4 (CTLA-4) antibody ipilimumab, have demonstrated clear ESRs and EERs.^13^ In contrast, data on the anti-programmed cell death protein 1 (PD-1) antibody nivolumab remain controversial, with some clinical reports showing convincing EERs in patients with non-small-cell lung cancer (NSCLC) and melanoma, whereas others failed to confirm this relationship.^14^^,^^15^ Of note, PK parameters such as drug clearance may be associated with the drug efficacy of nivolumab,^16^ not necessarily as a causal factor but rather as a predictor influenced by disease-related confounders, such as unfavorable tumor microenvironment or a hypercatabolic state in patients with cachexia, which can complicate the interpretation of EER.^17^ Regarding the PD-1-targeted antibody pembrolizumab, data from Merck suggest that plasma concentrations >10 μg/ml coincide with optimal target engagement.^18^ This was confirmed in a subsequent clinical report, indicating that patients with low trough levels of pembrolizumab were at greater risk of early progression.^19^ In line with this observation, a study in patients with NSCLC has suggested that PK-guided dosing could increase progression-free survival (PFS) compared with standard dosing, whereas for the anti-PD-(L)1 antibody, atezolizumab, PK modeling could instead help de-escalate dosing in a personalized fashion, possibly leading to a marked reduction in drug costs.^20^^,^^21^

For even more complex molecules, such as ADCs, understanding their EER and ESR features is particularly challenging because multiple forms (usually breakdown products) coexist in patients (i.e. intact ADCs, ADCs with reduced drug–antibody ratio, blank antibodies, free payloads, payloads attached to the linker or with further attachment to nanoalbumins).^22^^,^^23^ This complexity requires multiscale pharmacometrics. With sacituzumab govitecan [ADC targeting trophoblast cell-surface antigen 2 (TROP-2) and releasing 7-ethyl-10-hydroxycamptothecin, the active metabolite of irinotecan (SN-38) payload], the average plasma concentration (Cavg) of total antibodies (i.e. blank sacituzumab + sacituzumab govitecan) was associated with both PFS and overall survival. In contrast, the Cavg of sacituzumab govitecan alone was predictive of objective response rate (ORR) and toxicity.^24^ With trastuzumab–deruxtecan [ADC targeting human epidermal growth factor receptor 2 (HER2) and releasing deruxtecan], an EER has been found both in metastatic HER2-positive breast cancer and gastric cancer.^25^ Similar relationships with exposure have been observed with specific side-effects, such as interstitial lung disease. This suggests that TDM could be a valuable tool for predicting clinical outcomes, managing adverse effects in patients receiving ADCs (Figure 2), and helping with drug combinations.^22^

**Figure 2:**
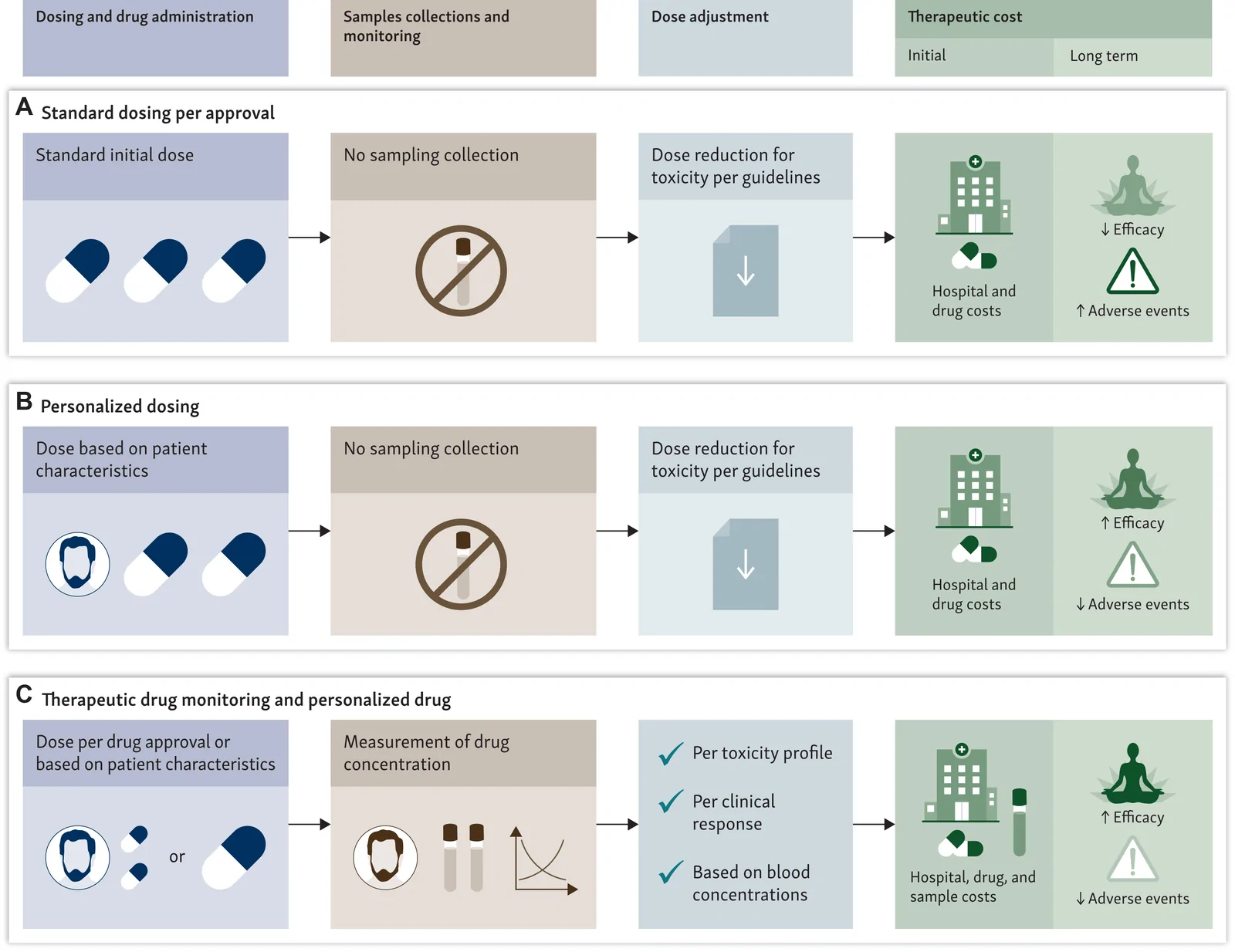
**Comparative strategies for dosing in oncology: standard, personalized, and therapeutic drug monitoring (TDM)-guided approaches.** This schematic compares three dosing strategies used in oncology: standard dosing, personalized dosing, and TDM with personalized dosing. (A) Standard dosing applies an officially approved dose (flat, weight-based, or body surface area-based) across the eligible per-approval patient population without initial dose adjustment based on the patient’s baseline characteristics. It can require adjustment when the toxicity observed exceeds initial expectations or there are suboptimal outcomes with an inefficient antitumor effect. Pros: reduced upfront costs (no monitoring), one treatment schedule for all eligible per-approval patients. Cons: can be responsible for treatment interruptions in the short and long term due to excessive or unmanageable toxicity or may overexpose some patients to overtreatment and undertreat others. Physicians are blinded to the exposure–efficacy ratio. (B) Personalized dosing tailors initial treatment based on patient-specific characteristics such as age, renal or hepatic function, and performance status. Pros: reduces toxicities, including long-term exposure-related ones, favors compliance, and spares pharmacological monitoring costs. Cons: lack of dynamic adjustment, exposing some patients at risk of undertreatment (with disease progression as a consequence) or overtreatment (increasing toxicities). (C) TDM-guided personalized dosing integrates initial personalization with ongoing measurement of drug concentrations in the blood, enabling dose adjustment based on pharmacokinetics. Pros: precise titration of therapy to achieve optimal drug levels, maximizing therapeutic effect while minimizing adverse events. Cons: at setup, costs are due to monitoring requirements (technical and skills). Figure created with BioRender.

Beyond dosing, personalized pharmacology provides insights into mechanisms of variability. Pharmacogenetic analyses, such as the detection of polymorphisms in dihydropyrimidine dehydrogenase (*DPYD*), UDP glucuronosyltransferase 1 family, polypeptide A1 (*UGT1A1*), or thiopurine methyltransferase (*TPMT*), helps to predict severe toxicities to fluoropyrimidines (*DPYD*), irinotecan and sacituzumab govitecan (*UGT1A1*), and thiopurines (*TPMT*), respectively.^26^^,^^27^ Although early identification can prevent severe toxicities, testing remains optional in many countries despite the availability of fast, inexpensive assays that outperform genotyping in sensitivity, as is the case for *DPYD* testing.^28^ Implementation of other upfront testing is highly variable, depending on the country and sometimes even between institutions within the same country.^29^

Drug–drug interactions affecting liver enzymes such as cytochrome P450 or membrane efflux transporters can markedly alter exposure to oral targeted agents, particularly in older or polymedicated patients.^30^ Lifestyle factors**,** including diet, smoking, alcohol consumption, herbal medicines, multivitamins, or acid-suppressive therapy, can further modulate drug absorption and clearance.^31^, ^32^, ^33^, ^34^ Despite their widespread recognition, these variables are seldom systematically integrated into clinical protocols or electronic decision-support systems.

Therefore, the pharmacological dimension of personalized medicine extends beyond biomarker stratification to the continuous optimization of drug exposure and patient safety across heterogeneous populations. As oncology moves toward individualized care, embedding pharmacology in early clinical development and routine practice will be critical to transform biological precision into ‘therapeutic precision’.

## Is it all about the target?

The identification of oncogenic drivers and tumor-specific molecular signatures has transformed cancer therapy from a histology-driven to a mechanism-oriented discipline. Molecular profiling now underpins treatment decisions for many tumor types, guiding the use of targeted agents against epidermal growth factor receptor (*EGFR*), anaplastic lymphoma kinase (*ALK*), *HER2*, or B-Raf proto-oncogene, serine/threonine kinase (*BRAF*) alterations, among others. Precision medicine, as the integration of tumor molecular background or oncogenic drivers, is routine for several tumor types, even though tumors are agnostic to the most potent drivers.^35^ Yet, despite the promise of precision, clinical experience reveals that ‘knowing the target’ does not always equate to ‘curing the disease’.

Although advances in genomic and transcriptomic technologies have enabled the discovery of oncogenic alterations, many of these alterations were, until recently, considered undruggable. One such target is Kirsten rat sarcoma virus (KRAS) proto-oncogene (KRAS) G12C, which has become druggable through advances in cysteine-targeted chemistry.^36^ KRAS G12C inhibitors have achieved clinical success in subsets of patients with NSCLC and colorectal cancer (combined with anti-EGFR monoclonal antibodies). Further investigations on more potent, less toxic brain-penetrant molecules targeting other *KRAS* mutations (KRAS G12D degraders) or even inhibiting PanRAS(ON), usually given in combinations, are ongoing.^37^ Recently, the multiselective active-state RAS (GTP-bound RAS) [RAS(ON)] inhibitor, daraxonrasib, showed efficacy both as a first-line treatment (in combination with gemcitabine–nab-paclitaxel) and as a single agent in previously treated metastatic pancreatic ductal adenocarcinoma, with a median survival of 13.2 compared with 6.7 months with standard chemotherapy.^38^^,^^39^ Another example includes *TP53*-mutated tumors, for which controversial roles in drug resistance have been reported, usually depending on the drug. Recent data on the first-in-class TP53 reactivator rezatopopt have shown impressive antitumor activity in heavily pretreated patients with advanced and metastatic tumors harboring the *TP53 Y220C* mutation, identified in ∼1% of all tumor types, with a meaningful ORR of 46% in heavily pretreated patients with platinum-resistant ovarian cancer.^40^^,^^41^

Artificial intelligence (AI) and high-throughput screening are accelerating this process by integrating structural modeling with real-world biological data to predict drug–target interactions more efficiently.^42^ The recently announced drug OKN4395, with the first patient dosed in a phase I trial assessing in solid tumors, was discovered based on target identification with AI.^43^ Together, these tools are reshaping target discovery into a data-driven discipline.^42^

However, the complexity of cancer biology challenges the notion that a single target can define treatment success. Resistance mechanisms, both intrinsic and acquired, inevitably emerge, driven by tumor heterogeneity, clonal evolution, and adaptive signaling networks. A striking example is seen with *EGFR*-mutated NSCLC: despite initial efficacy with first-generation (e.g. erlotinib or gefitinib) or third-generation inhibitors (e.g. osimertinib), secondary mutations, such as T790M or C797S, respectively, restore signaling.^44^ Moreover, signaling can be restored via bypass pathways, including mesenchymal-epithelial transition factor receptor (*MET*; also known as hepatocyte growth factor receptor, HGFR) amplification.^45^ This has led to the sequential development of generations of EGFR inhibitors and to combination strategies integrating bispecific antibodies (e.g. amivantamab and anti-MET-EGFR) or combinations with chemotherapy to overcome resistance.^46^, ^47^, ^48^ Similar dynamics are observed across other oncogene-driven tumors, where evolutionary plasticity continuously undermines the precision of single-agent therapies.

The need for dynamic monitoring has therefore become evident. Circulating tumor DNA assays now allow real-time assessment of sensitizing (e.g. for *EGFR* and *ALK-EML*) and resistance mutations and clonal evolution, enabling adaptive treatment decisions such as switching endocrine therapy in *ESR1*-mutated metastatic breast cancer, as demonstrated in the Palbociclib and Circulating Tumor DNA for ESR1 Mutation Detection (PADA-1) and Phase III Study to Assess AZD9833 + CDK4/6 Inhibitor in HR+/HER2-MBC With Detectable ESR1m Before Progression (SERENA-06) trials.^49^^,^^50^ Those studies strongly support a more proactive approach to overcoming therapy resistance by using molecular markers to guide treatment decisions. There was a lack of pharmacological exploration to ensure adequate treatment exposure at first-line treatment, especially for oral antihormonal drugs, considering that nonadherence to these drugs could be responsible for an increased risk of recurrence, as demonstrated with tamoxifen.^51^^,^^52^

Despite progress, most metastatic cancers remain incurable. Tumor evolution continues to outpace new treatments, revealing the limitations of relying solely on genomic strategies. Genuine personalization requires integrating molecular data with knowledge of pharmacodynamics, tumor microenvironment interactions, and host factors that influence treatment outcomes and side-effects. The field now recognizes that precision medicine should evolve into an ongoing process, constantly guided by biology, pharmacology, and individual patient circumstances. Ultimately, knowing how a drug works is only the beginning. Mechanistic clarity must be complemented by quantitative pharmacology**—**how exposure, drug accumulation and retention in the tumor, target engagement, and biological response interact in real patients. Translating these insights into optimized dosing, toxicity mitigation, and value-based care requires integrative PK and PD modeling supported by RWE. For example, several retrospective, real-world studies have suggested that administering immune checkpoint inhibitors in the morning is more effective than in the afternoon.^53^ Similar data on diurnal variation have been reported for conventional chemotherapy with 5-fluorouracil and oxaliplatin.^54^, ^55^, ^56^ The increased efficacy may be related to circadian variation in key enzymes involved in drug metabolism and DNA repair. The level of expression of circadian clock genes, like *PER3*, may play a role in the circadian variation in immuno- and chemotherapy.^56^, ^57^, ^58^

This transition from understanding ‘why’ a therapy works to determining ‘how best’ to use it forms the focus of the next section.

## Mechanistic insight as a foundation for precision cancer therapy and its challenges in access, reimbursement, and value assessment

The concept that drugs should preferentially act on diseased cells while sparing healthy tissues dates back to Paul Ehrlich’s ‘magic bullet’ hypothesis at the start of the 20th century. Although originally framed around selective targeting rather than modern mechanism-of-action biology, this principle anticipated a central goal of anticancer pharmacology: maximizing therapeutic effect while limiting toxicity. Early chemotherapy, exemplified by nitrogen mustards, succeeded largely through empirical observation rather than mechanistic insight.^59^ However, antifolates were the first group of drugs deliberately designed based on the understanding that folates are essential for tumor growth. Nonetheless, their clinical development was mostly empirical, even though childhood leukemia was initially selected as a fast-growing tumor. Yet, the later discovery of oncogenes, signaling pathways, and immune checkpoints fundamentally shifted cancer therapy from empirical approaches toward mechanism-informed treatment design. Today, understanding a drug’s mechanism of action is not merely academic: it determines how, in whom, and for how long a treatment should be used.

Mechanistic insight underpins every major success of modern oncology. Targeting HER2 overexpression in breast cancer with anti-HER2 therapies or inhibiting BRAF V600E in melanoma illustrates how decoding biological drivers enables transformative efficacy.^60^^,^^61^ However, these same examples reveal that mechanistic understanding must evolve with disease context: in colorectal cancer, BRAF V600E inhibition is ineffective without concomitant EGFR blockade, underscoring that pathway feedback and the tumor microenvironment dictate outcome.^62^ In breast cancer, a deeper understanding of the mechanism of action of trastuzumab identified its action beyond receptor blockade; it also forms a basis for antibody-dependent cellular cytotoxicity. This immune-mediated mechanism involves the recruitment and activation of immune effector cells that recognize and destroy HER2-expressing tumor cells.^63^ The interplay between trastuzumab’s direct inhibition of HER2 signaling and its ability to engage the immune system has contributed significantly to its therapeutic efficacy. Despite its success, a substantial proportion of patients either do not respond to trastuzumab or develop resistance over time, presenting a challenge in clinical management. Mechanisms underlying resistance may include alterations in HER2 signaling pathways, upregulation of compensatory growth factor receptors, or changes within the tumor microenvironment that diminish the effectiveness of trastuzumab.^64^ To address these challenges, several strategies have been developed or are under active investigation, including combination approaches, ADCs such as trastuzumab–deruxtecan, next-generation HER2-directed antibodies, including bispecific or biparatopic agents such as zanidatamab, and HER2-targeted tyrosine kinase inhibitors such as pyrotinib, tucatinib, and neratinib. The latter targets tyrosine kinase domains of multiple members of the HER2 family and has shown promise in overcoming resistance mechanisms associated with trastuzumab.^65^ The use of anti-HER2 therapies in routine treatment of early or metastatic breast cancer is a clear demonstration of precision medicine without personalization. Dissecting the HER2 heterogeneity with predictive tools, like HER2DX, can allow selection and prediction of the specific tumor subtypes that will benefit more from different treatment strategies.^66^^,^^67^

A further milestone has been the emergence of tumor-agnostic approvals**,** in which therapy is guided by molecular mechanism rather than histology. Drugs targeting neurotrophic receptor tyrosine kinase (*NTRK*) or rearranged during transfection (*RET*) fusions, microsatellite instability-high status, or high tumor mutational burden exemplify this shift from organ-specific to biology-defined treatment.^68^ These approvals showcase the triumph of mechanistic reasoning over morphology.^69^ Yet, they also expose profound regulatory and access disparities. Although agents such as larotrectinib and entrectinib received accelerated approvals from the FDA and European Medicines Agency, national reimbursement delays in several European countries have restricted patient access.^70^ The paradox is striking: a therapy scientifically justified by a universal mechanism may remain unavailable where it is most needed. Such gaps highlight the need for harmonized evaluation frameworks that weigh mechanistic validity and societal value alongside statistical evidence.^71^

For patients and advocates, understanding the mechanism translates into empowerment. It enables informed consent, clarifies the rationale for combination or de-escalation, and supports fair discussions on cost and value. Transparent communication of the mechanism of action and resistance mechanisms fosters trust and encourages shared decision making.^72^ Similarly, recognizing when the mechanism no longer offers benefit due to clonal evolution, microenvironmental adaptation, or PK insufficiency highlights the need for continuous monitoring and flexible treatment strategies.

## Optimizing oncology drug dosing and value assessments: the role of integrative modeling, novel trial designs, and RWE

Understanding how a drug should be used is as important as knowing how it works. Despite major advances in molecular precision, most anticancer drugs are still developed and prescribed using paradigms inherited from cytotoxic chemotherapy, such as dose escalation to a presumed MTD rather than to a biologically or pharmacologically optimal exposure. This approach often results in unnecessary toxicity, empirical dose reductions, and uncertain efficacy in broader patient populations. Bridging this gap requires an integrative pharmacological framework that couples mechanistic understanding with PK, PD, and real-world data. The key pharmacology-informed strategies discussed in this section are summarized in Table 1.

**Table 1:** Pharmacology-informed strategies for oncology dose optimization: applications, strengths, and limitations

Topic	Key problem addressed	Methodological contribution	Representative examples	Translational value	Main implementation challenges
Mechanistic PK/PD modeling	Empirical dosing and incomplete exposure–response understanding	Quantifies dose–ERRs and covariate effects	Imatinib, sunitinib, pembrolizumab, isatuximab	Supports rational dose selection and postapproval refinement	Requires robust exposure–response data, validated covariates, and pharmacometric expertise
MIPD	Interindividual variability in exposure and toxicity	Uses TDM, Bayesian forecasting, and patient-level covariates	Imatinib TDM, sunitinib adaptive dosing	Enables individualized adjustment after treatment initiation	Dependent on assay availability, turnaround time, software tools, and validated therapeutic thresholds
Alternative dosing (de-escalation and interval optimization)	Overtreatment, cumulative toxicity, and financial burden	Simulates lower-dose or extended-interval regimens while maintaining target exposure	Atezolizumab, ADCs, enfortumab vedotin	Reduces treatment burden while preserving efficacy	May reduce cost and toxicity but prospective clinical validation is often limited
Modern dose-finding trial designs	Limitations of MTD-driven 3 + 3 designs	Uses adaptive, Bayesian, CRM, and dose-ranging designs	Project Optimus-aligned early trials	Defines therapeutic windows rather than single tolerated doses	More complex to implement and may require larger early-phase datasets
Quantitative benefit–risk assessment	Difficulty integrating efficacy, toxicity, QoL and patient preference	Applies MCDA, clinical utility index, and patient-centered endpoints	Oncology dose optimization frameworks	Makes dose selection more transparent and patient-centered	Requires agreement on weighting efficacy, toxicity, QoL, patient preferences, and cost
RWE and causal inference	Limited generalizability of pivotal trials	Uses registries, RWE, g-methods, PBPK/PKPD, and hybrid modeling	Alternative sunitinib schedules, weekly docetaxel, imatinib TDM	Evaluates dosing in broader clinical populations	Vulnerable to confounding, missing data, heterogeneity in data quality, and regulatory constraints

ADC, antibody–drug conjugate; CRM, continual reassessment method; ERRs, exposure–response relationships; MCDA, multicriteria decision analysis; MIPD, model-informed precision dosing; MTD, maximum tolerated dose; PBPK, physiologically based pharmacokinetic; PD, pharmacodynamic; PK, pharmacokinetic; QoL, quality of life; RWE, real-world evidence; TDM, therapeutic drug monitoring.

### Modeling as a bridge between mechanism and clinical practice

PK/PD modeling provides a quantitative framework to link dose, exposure, biological activity, efficacy, and toxicity. In oncology, where many drugs have narrow therapeutic windows and substantial interpatient variability, these models can support dose selection, schedule optimization, and treatment individualization by integrating patient- and disease-related covariates such as age, sex, organ function, comedications, and genetic variability.^73^^,^^74^ Their clinical value, however, depends on the robustness of the underlying exposure–response relationship (ERR) and whether exposure targets are sufficiently validated to guide treatment decisions.

PK/PD modeling has contributed to several stages of oncology drug development and postapproval optimization. The EORTC-PAMM Group has historically contributed to optimizing drug-escalation strategies for conventional chemotherapeutics, including renal clearance-based models for carboplatin dosing.^75^ Similar PK modeling principles could be applied to targeted therapies such as osimertinib, which incorporate renal function as a covariate to guide dose adaptation and minimize toxicity.^76^ Model-informed approaches have also supported alternative dosing regimens for monoclonal antibodies and targeted agents, including isatuximab, belantamab mafodotin, roblitinib, and even pembrolizumab.^77^, ^78^, ^79^, ^80^ For immune checkpoint inhibitors, PK/PD modeling has contributed to the transition from body weight-based dosing to fixed dosing, extension of dosing intervals, and adaptation of regimens from adults to young adults and children.^18^^,^^81^, ^82^, ^83^, ^84^ Representative examples and their translational implications are summarized in Table 1. These cases underscore the role of plasma and tissue PK modeling in optimizing dosing strategies beyond initial regulatory approval.

### Innovations in dose (de-)escalation design and toxicity mitigation

Most de-escalation trials are based upon empirical and unilateral cut-in dosing, such as trials of immune checkpoint inhibitors. PK/PD modeling can be used to customize de-escalation, such as with atezolizumab, i.e. by fine-tuning dosing or dosing intervals to maintain drug levels above the threshold associated with target engagement.^21^ The extensive approval of ADCs based on unprecedented efficacy data raises concerns about the financial and long-term toxicity burden of those drugs.^85^^,^^86^ As an example, extensive modeling of enfortumab vedotin showed a cost reduction of 15%.^87^ However, the toxicity sparing of such an approach remains unclear. Long-term limiting toxicity by sensitive peripheral neuropathy remains challenging in this population of patients.^88^ Moreover, the approval of the enfortumab vedotin plus pembrolizumab combination for the first-line treatment of advanced or metastatic bladder cancer, replacing the longstanding use of cisplatin, leaves physicians with limited guidance on how to personalize therapy for individual patients, highlighting a gap in biomarkers and optimized treatment strategies for patient selection.^89^^,^^90^

The FDA established Project Optimus to modernize dose selection in oncology drug development by addressing significant shortcomings in dose selection strategies for anticancer therapies, particularly those revealed in recent clinical trials for targeted treatments.^91^ Two primary issues emerged. Firstly, in phase III trials, 30%-70% of patients required dose reductions due to intolerable toxicity, indicating that the doses determined in earlier, more controlled phases of development were unsuitable for the broader, more diverse real-world patient population.^92^ As stated previously, early-phase trials often include healthier, more homogeneous patient populations, while later trials and clinical practice involve individuals with greater variability in health and performance status. As a result, clinicians frequently resort to empirically reducing doses to mitigate toxicity but this practice introduces uncertainty about whether these empirical lower doses retain their therapeutic efficacy. Secondly, for many newer therapies, such as targeted agents and novel treatment modalities, the dose–response curve often plateaus, meaning that increasing the dose beyond a certain point provides minimal additional therapeutic benefit while significantly increasing toxicities.^92^ These challenges underscore the need for strategies aligning dose selection with efficacy and tolerability across diverse patient populations. Through the evaluation of alternative dosing regimens and the early integration of both safety and efficacy considerations in drug development, Project Optimus seeks to optimize the therapeutic potential of emerging treatments by improving dose selection with a goal of ensuring that novel therapies are safer and more effective, improving the transition into clinical practice, and ultimately enhancing patient outcomes.

Traditional dose-finding strategies in oncology, particularly the 3 + 3 design, remain widely used despite their limitations.^93^^,^^94^ Originally designed for cytotoxic chemotherapy, this approach assumes a direct relationship between toxicity and efficacy, an assumption that does not always hold for targeted therapies and immuno-oncology agents. MTD, often determined by acute toxicities, may not reflect long-term tolerability or optimal therapeutic benefit,^95^ especially because Common Terminology Criteria for Adverse Events grading by investigators and actual PROs may differ.^96^ Alternative strategies, including Bayesian adaptive design trials and continuous reassessment methods, offer a dynamic approach in which doses for subsequent participants are adjusted using Bayesian inference or model-based algorithms based on prior data.^95^ Additionally, a growing focus on characterizing full dose–exposure–response curves is shifting the paradigm from identifying a single ‘optimal’ dose to defining a therapeutic window that balances efficacy and safety. Unfortunately, dose-ranging trials remain limited, with only 20% covering a broad enough range to assess ERRs extensively.^97^

Quantitative benefit–risk assessment frameworks, such as the clinical utility index and multicriteria decision analysis, are emerging as valuable tools for integrating efficacy, toxicity, and patient-centered outcomes into dose selection.^98^^,^^99^ These approaches, combined with PK/PD modeling, enable more data-driven and individualized dosing strategies (Figure 3).

**Figure 3:**
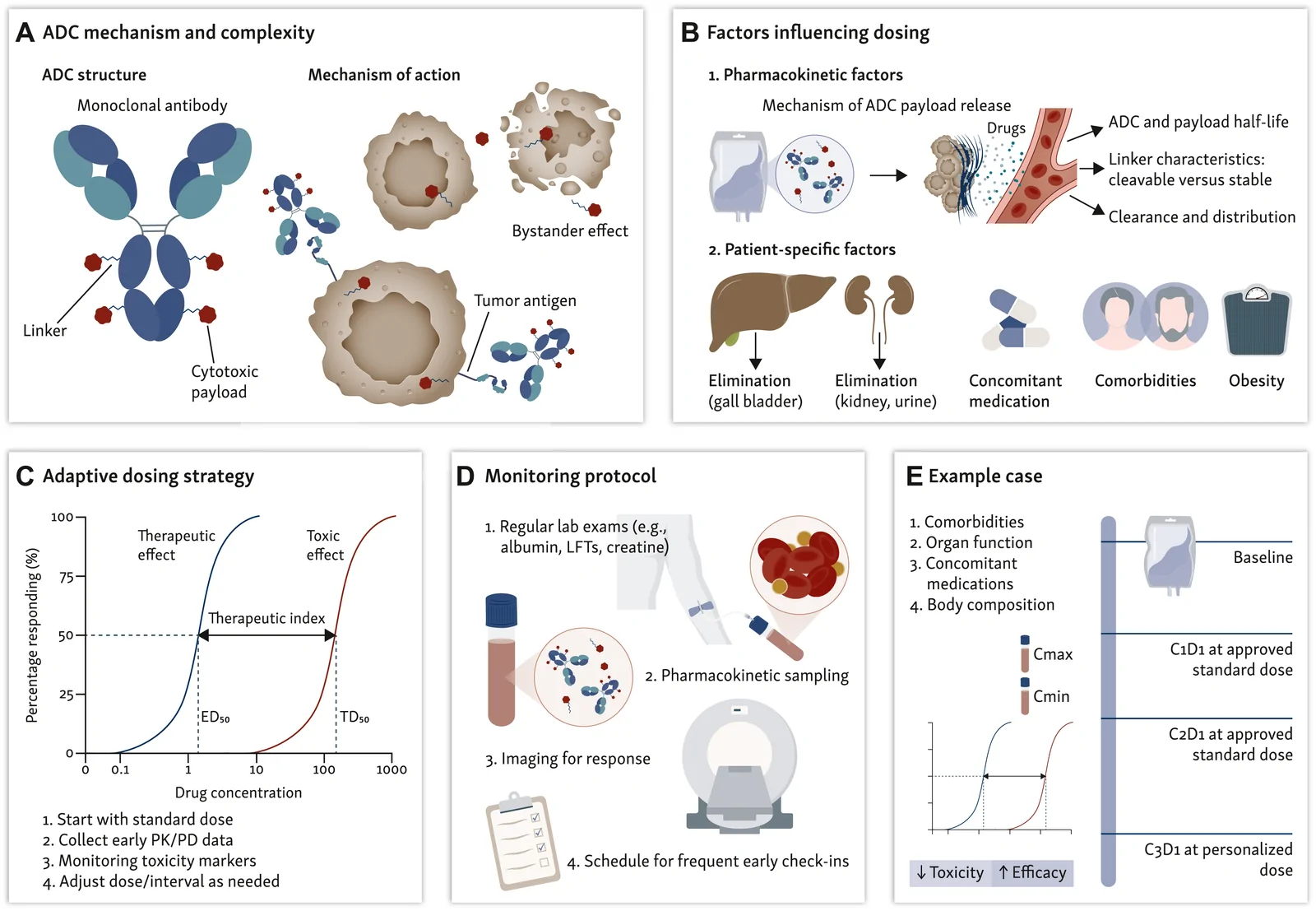
**Framework for individualized dosing optimization in ADC therapy.** This figure illustrates how to optimize ADC dosing based on drug, patient-specific factors, and dynamic response monitoring, including monitoring of circulating tumor DNA. (A) An ADC contains antibodies, linkers, and cytotoxic payloads. The way the ADC is built impacts its mechanisms of action: binding to tumor antigen on the tumor cell surface, ADC internalization, release of cytotoxic payload with or without a linker (depending on the conjugation used), and release of cytotoxic payload in the extracellular space, either by payload detachment from the ADC or by its release from dying cancer cells. Several parameters impact the ADC mechanism of action: variability in antigen expression, internalization rates, linker stability, and payload resistances, which contribute to interpatient drug exposure and response differences. (B) Dosing decisions are influenced by PK factors (e.g. ADC and payload half-life, drug clearance, linker stability, conjugation type) and patient-specific variables (e.g. liver and renal function, concomitant medications, comorbidities, and body composition). (C) An adaptive dosing strategy begins with a standard dose and is adjusted based on early PK/PD data and toxicity markers. (D) A structured monitoring protocol enables close monitoring of safety profiles based on patient-reported adverse events and biological abnormalities, tumor response with imaging and biological sampling whenever possible, and dose adjustment based on laboratory tests, PK sampling, and imaging. (E) An example case highlights how dose adaptation is used to adjust ADC dosing. The patient’s-specific baseline characteristics are considered. Modeling of drug exposure is based on ADC monitoring of plasma samples (the entire ADC, antibody, and/or payload, depending on the ADC construction and properties), with sampling collection at Cmax (timepoint depending on the ADC type) and the residual plasma concentration measured from the predose PK sample collected just before the cycle 2 infusion. The model is run before cycle 3 and the dosing can be adapted accordingly. The final goal is to decrease side-effects, especially in patients with long-lasting exposure, and to gain efficacy by readapting the dosing to the tumor response. ADC, antibody–drug conjugate; C1D1, cycle one day one; Cmax, maximum concentration; Cmin, residual concentration; LFT, liver function test; PD, pharmacodynamic; PK, pharmacokinetic. Figure created with BioRender.

Lastly, biomarker-driven dose optimization remains underused but holds promise for refining dosing beyond empirical escalation. Tumor- and safety-related biomarkers, particularly those linked to immune-related toxicities, could guide more precise dosing and improve patient outcomes.^100^^,^^101^ Despite challenges, including validation, standardization, and regulatory acceptance, integrating mechanistic insights, preclinical data, and PK/PD modeling into dose selection could enhance precision oncology and improve therapeutic decision making. In the Investigation of Profile-Related Evidence Determining Individualized Cancer Therapy (I-PREDICT) program, personalized treatment regimens, almost N-of-1, were proposed to all patients, including previously unassessed combinations, driven by molecular alteration. Dose finding was guided by patient toxicities with progressive interpatient dose escalation.^102^ However, no PK modeling was applied.

AI further amplifies this potential by enabling multiscale analysis of complex biological and clinical data, facilitating the identification of optimal dosing strategies tailored to propose customized dosing.^103^ By integrating AI-driven insights with forward-looking regulatory initiatives like Project Optimus, the development and delivery of next-generation cancer therapies can be significantly accelerated.

### Narrowing the gap between clinical studies and practice

Regulatory agencies are increasingly leveraging RWE to support drug approvals, label expansions, and postmarket safety monitoring.^104^ One of its key applications is addressing evidence gaps for underrepresented populations, such as elderly patients, pediatric patients, and individuals with comorbidities.

Advanced causal inference methods, including the parametric g-formula and physiologically based PK modeling, allow for the extrapolation of trial data to real-world settings. Whereas causal inference excels with large datasets, PK/PD modeling provides mechanistic insights with smaller sample sizes. Thus, merging the two approaches could enable causal inferences even from the smaller datasets typically available in clinical trials.^105^ Additional hybrid approaches, incorporating agnostic machine learning strategies with mechanistic modeling, such as quantitative system pharmacology, are emerging to optimize dose–response predictions and treatment personalization.^106^

High-quality RWE collection is essential for successful integration into clinical practice. This can be extended to demonstrate the benefit of TDM and adaptive dosing strategies for drugs once approved with a flat dosing schedule, such as with imatinib. From pivotal trials, led by the industry, this drug was initially approved at 400 mg once daily dosing. Independent academic groups have demonstrated that trough levels of 1000 ng/ml were associated with major molecular response and that adaptive dosing could help to improve clinical outcomes in real-world patients.^107^ In gastrointestinal stromal tumors, the 2023 Spanish Group for Sarcoma Research guidelines further endorse measuring imatinib plasma levels in cases of unexpected disease progression despite standard dosing, although recent research highlights the practical challenges that still hinder the routine use of TDM- and pharmacogenetic-guided dose individualization.^108^ Further studies have identified several baseline covariates, such as body mass index, that are likely to eventually modify patients’ PK parameters and exposure levels, suggesting that they could be used to customize dosing at treatment initiation.^109^

Another example of how routine practice can lead to substantial change in dosing strategies is sunitinib, initially approved at 50 mg once daily dosing with a 4-weeks-on, 2-weeks-off schedule. RWE has suggested that shifting to 37.5 mg or keeping the 50 mg once daily dosing but with an alternative 2-weeks on, 1-weeks off schedule would be better tolerated without compromising efficacy.^110^ Similarly, canonical cytotoxic docetaxel is still officially approved as 90 mg/m^2^ every 3 weeks in breast cancer. In contrast, routine practice has suggested that lower 25-40 mg/m^2^ weekly dosing is a promising approach in the metastatic setting.^111^ Of note, PK/PD modeling of sunitinib in gastrointestinal stromal tumors indicated no additional clinical benefit from higher exposure, supporting standard dosing strategies and avoiding unnecessary adjustments.^112^ Although high sunitinib concentrations showed increased toxicity in a number of cancer cell lines, high-dosing scheduling was ineffective in clinical studies.^113^ In metastatic renal cell carcinoma, TDM coupled to Bayesian adaptive dosing of sunitinib helped restore efficacy, including in patients with initially inadequate exposure when treatment started with approved dosing.^114^

Despite a strong biological rationale and growing clinical evidence supporting these methods, various practical and structural barriers continue to hinder their widespread use in routine oncology practice. Implementing PK/PD modeling, TDM, and MIPD requires dedicated infrastructure, including access to validated bioanalytical assays, specialized software, and smooth integration into clinical workflows. Turnaround times for PK analyses may not be compatible with real-time clinical decision making in many settings. Additionally, these strategies rely on specific expertise in clinical pharmacology and pharmacometrics, which remains unevenly distributed among institutions. Economic factors, such as assay development and validation, modeling platform, and personnel costs, along with uncertain reimbursement policies, further limit adoption. Notably, the absence of standardized, prospectively validated exposure targets across many drug classes limits the clinical applicability of these approaches. Some ERRs are robust for some agents but less reliable or actionable for others. Overall, these challenges emphasize the need for prospective validation, methodological harmonization, and health system support to widen the use of precision dosing strategies in oncology.

Collaborative efforts, including oncology-specific registries and randomized registry trials, improve longitudinal data capture and real-world treatment evaluation.^115^ For example, Scandinavian countries have introduced national health registry development, offering models for systematic data collection to inform evidence-based oncology care. Expanding such initiatives globally will be important for optimizing cancer therapy outcomes in real-world settings and oncology-specific registries are now being developed in the UK, the Netherlands, and the European Network of Cancer Registries.^116^ Although those initiatives show a trend toward comprehensive data collection, at the European level, some legislation limitations require innovative approaches from policymakers.

## Conclusion

The growing complexity of cancer therapeutics reflects not only the expanding arsenal of targeted and immune-based agents but also the intrinsic biological complexity of cancer itself as a heterogeneous, evolving disease embedded within a dynamic host system. Advances in genomics, immunology, and bioengineering have reshaped oncology into a mechanism-driven discipline. However, clinical success still depends on how effectively mechanistic knowledge captures tumor evolution, host–tumor interactions, and treatment-induced adaptation over time and how these insights are translated into personalized therapy. Personalized oncology is therefore not achieved by target selection alone; it requires the integration of pharmacology, clinical science, and patient-centered evidence.

Despite decades of progress, most anticancer drugs continue to be developed and prescribed using empirical dosing paradigms that ignore interpatient variability. Integrating PK and PD principles into every stage of drug development can bridge this gap. By quantifying how exposure relates to efficacy and toxicity, model-informed approaches can refine dose selection, improve trial design, and reduce the high attrition rate in oncology. Equally, TDM and adaptive dosing strategies in routine care can ensure optimal exposure for diverse populations, including those historically underrepresented in clinical research.

However, scientific optimization must go hand in hand with social responsibility. The emergence of tumor-agnostic therapies, although a success of mechanistic reasoning, also highlights disparities in national approval and reimbursement processes. Ensuring equitable access to these innovations requires evidence frameworks that integrate mechanistic validity, patient heterogeneity, and value-based assessment. Pharmacology plays a pivotal role to play in this dialogue by providing objective metrics for efficacy, safety, and cost effectiveness to inform regulatory and policy decisions.^117^

The future of personalized oncology lies in the convergence of mechanistic insight, quantitative pharmacology, and RWE. Together, these disciplines enable a continuous feedback loop, from preclinical modeling to bedside monitoring, transforming oncology into a learning health system that adapts to each patient and context. Realizing this vision demands closer collaboration between pharmacologists, clinicians, data scientists, and patient advocates.

As a multidisciplinary network committed to bridging pharmacology and clinical oncology, the EORTC-PAMM Group advocates for this integrative model. Embedding pharmacological principles across the drug development continuum can enhance both efficacy and safety while ensuring that the benefits of innovation are distributed equitably and sustainably. Personalized medicine must therefore evolve from a promise of molecular precision to a practice of measured, equitable, and data-driven care—bringing us closer to the goal of fully optimized oncology.
